# Activity of Organoboron Compounds against Biofilm-Forming Pathogens

**DOI:** 10.3390/antibiotics13100929

**Published:** 2024-09-29

**Authors:** Monika I. Konaklieva, Balbina J. Plotkin

**Affiliations:** 1 Department of Chemistry, American University, 4400 Massachusetts Ave. NW, Washington, DC 20016, USA; 2 Department of Microbiology and Immunology, Midwestern University, 555 31st St., Downers Grove, IL 60515, USA; bplotk@midwestern.edu

**Keywords:** boron-containing compounds, antimicrobial, anti-biofilm, multidrug resistance, inhibitors of pathogenic bacterial enzymes, phenotypic screens

## Abstract

Bacteria have evolved and continue to change in response to environmental stressors including antibiotics. Antibiotic resistance and the ability to form biofilms are inextricably linked, requiring the continuous search for alternative compounds to antibiotics that affect biofilm formation. One of the latest drug classes is boron-containing compounds. Over the last several decades, boron has emerged as a prominent element in the field of medicinal chemistry, which has led to an increasing number of boron-containing compounds being considered as potential drugs. The focus of this review is on the developments in boron-containing organic compounds (BOCs) as antimicrobial/anti-biofilm probes and agents.

## 1. Introduction

**Figure 1:**
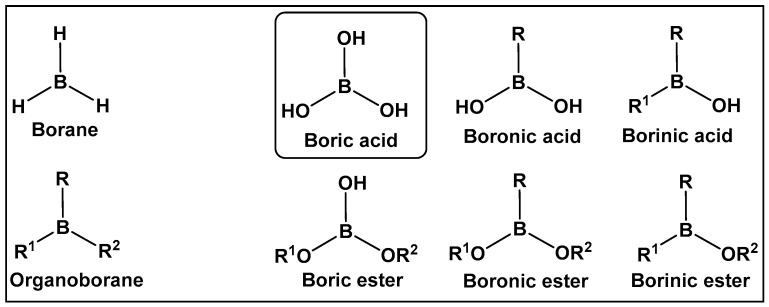
Introduction to the classes of boron-containing compounds: borane, organoboranes, oxygen-containing boron compounds. To date, fully oxygenated boric acid (BA) is the only naturally occurring form of boron reported.

**Figure 2:**
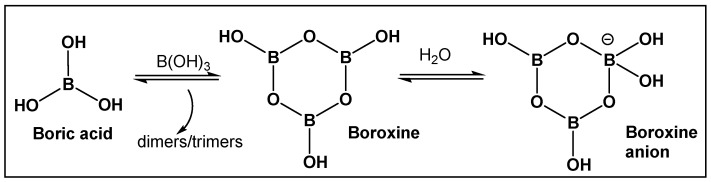
Boric acid and its linear and cyclic forms as a result of self-assembly and polymerization with additional boric acid molecules.

Boron-containing organic compounds. In the periodic table, boron is to the left of carbon, and thus its electronic proximity to carbon has drawn comparisons including the highlighting of their different roles in nature [1]. Both carbon and boron show considerable specialization, as compared to the vast majority of other chemical elements [2]. This functional focus correlates with boron being differentially sequestered across the different organismal domains, due to its ability to dynamically interact with organic molecules. Boron is an exception to the octet rule, in that it is stable with fewer than eight valence electrons. Boric acid (Figure 1 and Figure 2) is the only naturally existing form of boron. It can undergo self-assembly and polymerization to give rise to linear and cyclic hydroxyborons (Figure 2). The only process currently known through which the living organisms prepare boron-containing organic compounds is borylation, that is, the insertion of hydroxyborons [B(OH)n] available in the given organisms’ environment (marine/soil) into predominately small organic molecules [3,4,5,6,7,8,9,10]. Boron is typically incorporated into hydroxy- or amino-rich domains, i.e., those with oxygen/nitrogen Lewis Bases, of a given structure Its presence appears to lead to endogenous functional effects that are unavailable through the known types of “main” bonding in biological systems, based on the organic chemistry of carbon, hydrogen, nitrogen, and oxygen [11].

Organisms that incorporate boron into organic compounds via boron–oxygen bonds, are very limited; however, in nature, compounds with carbon–boron bonds (e.g., organoboranes) have not been reported. Until several years ago, the syntheses of organoboranes were the sole prerogative of organic synthetic chemists [12,13,14]. That has changed with the utilization of enzyme-catalyzed borylation leading to the preparation of chiral organoboranes by a genetically encoded platform in *Escherichia coli* [15]. Since the 1990s, especially following the pioneering work of Suzuki and Miyaura on the utilization of organoboron compounds for C-C bond formation resulting in the production of conjugated systems of alkenes, styrenes, or biaryl compounds, the publications of both synthetic methodology and drug development of boron-containing structures have skyrocketed [16]. The synthetic methodology has paved the way for the use of new boron-containing organic molecules with pharmaceutical applications, e.g., boronic acids and their derivatives. The air-stable organic sp^2^ boron compounds with a neutral hybridized trivalent boron, such as in boronic acids, have a center with a vacant p orbital. The latter is the reason for the mild Lewis acidic character displayed by boronic acids. They, and other boranol (B-OH)-containing compounds, due to their ability to form tetracoordinate conjugate base (sp^3^ hybridized) in water, display indirect Brønsted acidity The Lewis acid character and the ability to convert between sp^2^ and sp^3^ hybridized boron–hydroxy complexes enable boronic acids/hemiboronic acids, e.g., benzoxaboroles, to participate in a variety of covalent and hydrogen bond interactions with biological membranes/target proteins at physiological conditions thus placing them in the drug class of reversable covalent inhibitors.

There are many excellent in-depth reviews on boron chemistry, and the reader is directed to several excellent research articles and reviews summarizing various aspects of the current state of the synthetic methodology and drug development of boron-containing organic molecules [17,18,19,20,21,22,23,24,25]. The limited number of boron-containing organic molecules found in nature has left chemists with very few natural leads as models for drug development. That differs dramatically from the number of the rest of the known naturally occurring antimicrobials, and perhaps that provided the opportunity for the “de novo” synthesis and use of organoborons as medicinal agents. This review outlines the rationale for boron-containing organic compounds as antimicrobials/anti-biofilm agents. The bibliography contains 216 references from the early examples of organoboron as antimicrobials/anti-biofilm agents (1960s) to the first half of 2024. 

A biofilm is a population of microbes that attaches itself to biotic and abiotic surfaces, while producing extracellular polymers to encase itself for protection against environmental assaults. The vast majority of chronic infections and environmental biofouling issues are the result of biofilm formation. Boron-containing small organic molecules are being explored as anti-biofilm compounds, and some have demonstrated promising activity in interfering with the biofilm formation of bacteria and fungi. Historically, they have been developed as inhibitors of the established antimicrobial drug targets, such as β-lactamases. The discussion of the latter is included herein, due to their role as a starting point for their use as anti-biofilm agents. Polymeric structures, utilizing the ability of boron to stabilize crosslinks in these macromolecules, or its binding interactions with different moieties of the polymeric structures of the bacterial surface (e.g., membranes), have been the focus of exploration as anti-biofilm agents, as well and their antimicrobial and antifungal properties are also included herein.

Biofilm. It is estimated that more than 80% of all microbial infections involve biofilm formation. In the last two decades, it has been established that the formation of biofilm is the predominant life-mode of most bacterial species [26]. The studies of the molecular mechanisms underlying biofilm formation and biofilm dispersal have led to the identification of distinct molecules and mechanisms as targets for drugs specifically designed to function as anti-biofilm agents. The molecular target of each anti-biofilm drug class is described below.

The main target for many boron-containing molecules is quorum sensing (QS), a regulatory process that has a role in biofilm formation and is an overall regulator of virulence factor expression in pathogenic bacteria [27]. The development of specific anti-biofilm drugs is a strategy that is expected to alleviate the current antibiotic crisis due to the development of drug resistance. It should be noted that antimicrobial resistance (AMR) alone will add an expected annual cost of $100 trillion in the USA alone by 2050 [28]. Potential anti-biofilm drugs could be classified as next-generation antimicrobials (NGA) since the target, i.e., biofilm formation, is different from the existing targets of the currently used clinically relevant antibiotics. Ideally, NGAs should also possess anti-virulence properties at concentrations that do not impact bacterial viability, thereby minimizing the selective pressure they apply and the probability of developing bacterial resistance towards them [29].

## 2. Utilizing the Boric Acids’ Scaffold by Following Nature’s Pragmatism

### 2.1. Naturally Occurring Bacterial Boron–Oxygen Bond-Containing Compounds (Boric Acids and Derivatives)

#### 2.1.1. The Unique Antimicrobials Containing Boron

**Figure 3:**
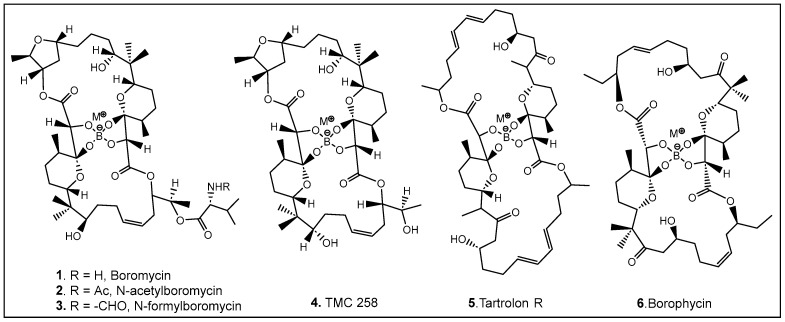
Representative examples of currently known boron-containing antibiotics forming a Böeseken complex (a complex formed between alcohols/α-hydroxy acids and boron) [51,53].

The most common naturally occurring boron-containing molecules are the complexes of boric acid with the diol moiety of biomolecules. About 6 decades after the discovery of the importance of boron as microelement for higher plants [30,31,32,33,34,35,36,37,38], the first organic compound containing boron was reported. Boromycin **1** (Figure 3) was isolated from the *Streptomyces* species [3,5,39,40,41]. A structurally similar compound named tartrolon **5** (Figure 3) [42,43] was subsequently isolated from *Sorangium cellulosum*, a myxobacterium [8] and from the symbiotic cellulose-degrading bacteria in shipworm gills [5]. Derivatives on the amino group of boromycin N-acetyl- (**2**, Figure 3) and N-formyl- (**3**, Figure 3) boromycin have also been isolated from the boromycin-producing *Streptomyces antibioticus* [4], as well as another boromycin derivative—desvalinoboromycin or TMC 25B **4** (Figure 3) from soil *Streptomyces* spp. [44]. Boromycin, together with several other polyketide antibiotics (Figure 3), belongs to the family of the boron-containing antibiotics produced by Gram-negative bacteria, which were initially found to be effective against Gram-positive bacteria [3,4,5,41]. Several other activities of boromycin and its derivatives were later determined, i.e., antiviral, specifically anti-HIV [44,45], coccidiostatic [46], anti-toxoplasmitic/anti-cryptosporidic [41,47], and anti-mycobacterial [48]. Boromycin, a K^+^ ionophore [48], has also been found to have effect on Ca^2+^ homeostasis [49]. In boromycin and structurally related antibiotics, boron plays a structural role, inducing the folding of the polyols into compact structures [17]. The potent cytotoxin, borophycin, was isolated from the marine cyanobacteria *Nostoc spongiaeforme* var. *tenue* [50] and *Nostoc linckia* [51] and hasbeen investigated for its anticancer activity [52]. The structural elucidation, biosynthesis, synthesis, and biological activities of boron-containing antibiotics have been extensively reviewed [9,10,53,54].

#### 2.1.2. Autoinducer-2 (AI-2) Quorum Sensing (QS) Inducer: A Borate Diester

**Figure 4:**
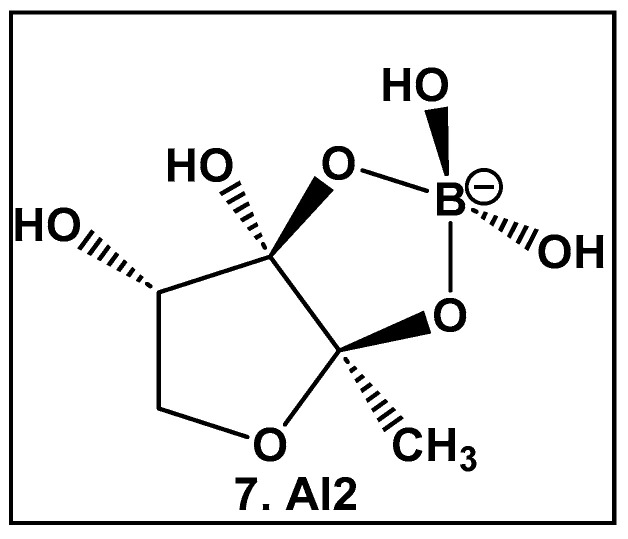
Structure of AI-2, the universal QS autoinducer: a boron-diester.

AI-2 furanosyl borate diester complex **7** (Figure 4), which highly resembles the core of the larger boron-containing antibiotics (Figure 3), was isolated from the bioluminescent marine bacterium *Vibrio harveyii* as one of two autoinducers that regulate light production in this bacterium in response to cell density (quorum molecule) [55,56]. Bacteria communicate using extracellular (hormone-like) signal molecules termed as autoinducers in a process called quorum sensing, which is typically dependent on the cell population size. It has been found that both Gram-negative and Gram-positive bacteria have the necessary enzymatic machinery to produce AI-2 [57]. Thus, the boron-containing AI-2 has been suggested to be the “universal” bacterial QS signal in interbacterial communication [55,56,57,58,59,60,61,62]. That realization has led to the design and synthesis of AI-2 analogs [60,61], as well as various potential AI-2 inhibitors [63,64,65,66,67,68,69,70,71,72]. To date, there are no FDA-approved drugs as AI-2 autoinducer inhibitors, most likely due to the complexity of QS signaling and promiscuity of the receptors which are involved in the synergistic agonism of AI-2 and analogs [62].

#### 2.1.3. Rhamnogalacturonan II (RG-II)—a Boron–Sugar–Alcohol Complex

**Figure 5:**
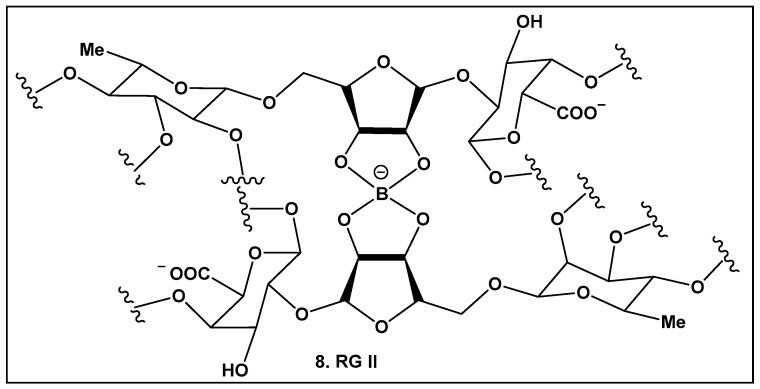
Boron-containing compounds in higher plants: Partial structure of Rhamnogalacturonan II (RG-II) with focus on boron–oxygen complex at the center of the RG-II molecule.

Boron carbohydrate complexes have been isolated from bacteria, seaweed, lichens, and fungi [9,10], with boron alcohol complexes from higher plants discovered close to half a century ago [73]. Their representative, Rhamnogalacturan II (RG-II) (**8**, Figure 5), is one of the most complex carbohydrates ever found in nature [73]. RG-II is found in the primary cell walls of all vascular plants [74,75,76,77,78,79]. The borate crosslinking in this carbohydrate is required for the normal plant development and growth, and its production requires an array of different proteins [74,75,76,77,78,79,80,81]. Mutations leading to alterations in the RG-II structure also affect crosslinking and could lead to severe impaired growth or even to plant death [82]. Beyond plants, RG-II has been recently identified as a Toll-like receptor 4 agonist that inhibits tumor growth by activating dendritic cell-mediated CD8^+^ T cells [83] in mammals.

Regardless of the type of organisms in which they are present, natural boron-containing compounds are those with boron–oxygen bonds. The rapid improvement in many spectroscopy techniques, such as nuclear magnetic resonance (NMR) and, more specifically, Boron11 NMR, has allowed for the detection of a higher percentage of naturally occurring boron-containing compounds than expected [84]. It will be interesting to see whether the yet to-be-discovered boron-containing compounds will have more variety in boron-bonding, or whether they will confirm nature’s pragmatism in binding.

## 3. Compounds Prepared by Organic Syntheses: Not Yet Found in Nature

### 3.1. “Nonclassical” Anti-Bacterial Chemotypes: Synthetic Boron—Carbon Bond-Containing Compounds

**Figure 6:**
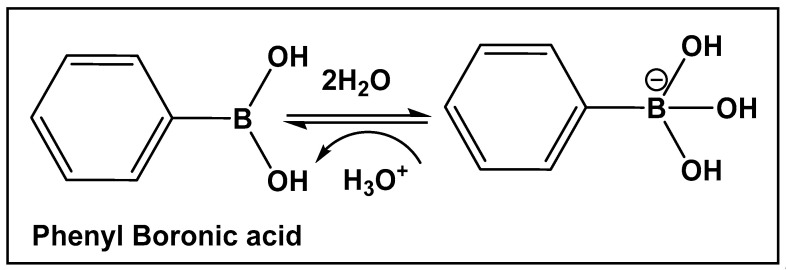
Illustrative example of the interconversion of the hybridization of boronic acids in the presence of water.

Boron-carbon bond-containing compounds’ reversible interconversion from sp^2^ to sp^3^ in the presence of nucleophiles is shown in Figure 6.

#### 3.1.1. Boronic Acids and Their Cyclic Hemiesters: β-Lactamase Inhibitors

**Figure 7:**
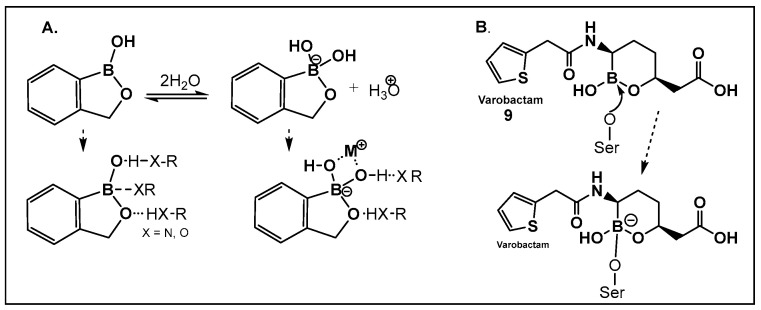
(**A**). Equilibrium dissociation and biologically relevant interactions of benzoxaborole in water; (**B**). Molecular mode of inhibition of vaborbactam, **9**, a hemiboronic acid drug by the active site serine.

**Figure 8:**
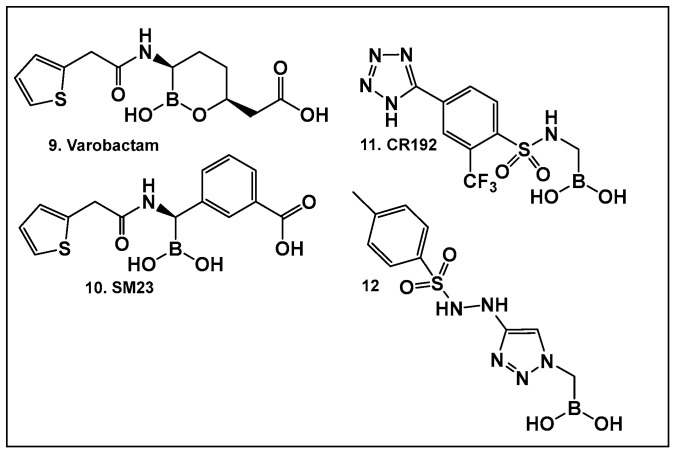
Examples of boronic acids and their hemiesters targeting β-lactamses: Boronic acids **10**–**12** and the cyclic hemiester of boronic acid, and FDA-approved Vaborbactam **9** as β-lactamase inhibitors.

Vaborbactam (**9**, Figure 7 and Figure 8) is a boron-containing compound approved by the FDA (2017) as a β-lactamase inhibitor [85]. It is considered the most potent inhibitor of its class, with activity against class A serine carbapenemases. Specifically, vaborbactam’s inhibitory function is restricted to *Klebsiella pneumoniae* carbapenemase (KPC), while exhibiting no activity against mammalian serine proteases. The potential of boron-containing compounds and specifically that of boronic acids (BAs) as antimicrobials was demonstrated in the 1970s, with the use of the boronic acid (BA) as a reversible inhibitor of the serine β-lactamase from *Bacillus cereus* [86]. The hydrolysis of β-lactams by the β-lactamases involves the well-documented formation of a tetrahedral intermediate. Boronic acids are often described as “transition state analogs” (BATSIs), since they have a Lewis Acid (LA) property, i.e., they can act as electrophiles, a quality that permits the formation of tetrahedral adducts with alcohols/(serine) enzymes by interconversion between their sp^2^ and sp^3^ forms (Figure 7) [85,86,87,88,89,90,91,92,93]. Securing target selectivity, i.e., maximizing the selectivity for a given enzyme, e.g., β-lactamases, leading to diminishing/evading the off-target effects, is of great importance for the success of a given structure as a drug candidate. 

The utilization of α-aminoalkyl boronic acids, classified amongst the α-amino acid analogs, led to the synthesis of peptidyl derivatives of the boronic acid as selective β-lactamase inhibitors. These included SM23, **10** (Figure 8) which has activity against *Acinetobacter baumannii* cephalosporinase (ADC-7, Ki 21 nM) [94,95,96], as well as class C β-lactamase (AmpC, Ki 1nM) [88,89,90]. SM23 **10** exhibits an improved inhibition (>2-fold) as compared to the analogous glycylboronic acids [90], including RPX7009, (Vaborbactam) **9**, (Figure 8). Preparation of a cyclic boronic acid derivative RPX7009, (Vaborbactam) **9**, (Figure 8) led to high potency and selectivity for the β-lactamases vs. the human proteases, which have an affinity to acyclic substrates/molecules [85]. Vaborbactam has been approved by the FDA for use in combination with meropenem to selectively target pathogens producing serine carbapenemases [93,94]. This narrow FDA usage approval is despite its inhibitory activity against many other types of serine β-lactamases [94], with it demonstrating particularly potent activity against class A enzymes, *Klebsiella pneumoniae* carbapenemase (KPC), extended spectrum β-lactamases (ESBLs), Cefotaximase-Munich (CTX-M), sufhydryl reagent variable (SHV), and class C cephamycin β-lactamase (CMY) enzymes [91,92,93,94,95,96]. In addition, it has better activity as compared to the β-lactamase inhibitors clavulanic acid and tazobactam against the class A carbapenemase KPC-2 (Ki 0.069 mM), as well as the class C enzymes P99 and CMY-2 (Ki 0.053; Ki 0.03 mM, respectively) [85]. Both SM3, **10**, and RPX7009 (Vaborbactam), **9** (Figure 8), activities are based on an amide scaffold, which gives them the ability to mirror the β-lactams’ amide chain, a structural feature that makes them highly selective and potent as β-lactamase inhibitors. 

The replacement of the amide moiety with sulfonamide as in CR192, **11** (Figure 8), [95] was intended to make the structure a better fit for the β-lactamase active site. Amide replacement with its non-classical bioisostere—1,2,3-triazole as in **12** (Figure 8)—was explored to prepare both lactamase inhibitors and molecular probes [96]. Both compounds in CR192, **11** (Figure 8, Ki 0.45 nM) [95] and **12** (Figure 8, Ki 90 nM for ADC-7) [96], demonstrated β-lactamase activity better than Vaborbactam (Ki 0.72 mM) against that enzyme.

**Figure 9:**
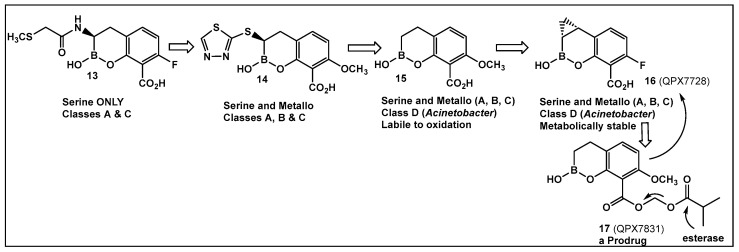
Evolution of the hemiboronic acids toward broadening their spectrum as β-lactamase inhibitors (serine- and metallo-β-lactamases) and improving their metabolic stability [97,98,99,100].

Further efforts toward both broadening the spectrum and improving the metabolic stability of vaborbactam have led to the synthesis of QPX7728 (**16**, Figure 9). The latter has a broad spectrum of inhibition, including class B and class D enzymes, and is considerably resistant to porin modifications and efflux [97]. The class B (metallo) enzyme NDM-1 of *Enterobacteriaceae* and the class D (OXA) enzymes of *Acinetobacter baumannii* continue to be refractory to inhibition, despite the success of vaborbactam as a broad-spectrum β-lactamase inhibitor. Therefore, the discovery of cyclic boronic acid QPX7728 (**16**, Figure 9), an ultra-broad-spectrum inhibitor of serine and metallo-β-lactamases, is a very important advancement in the development of β-lactamase inhibitors [97]. Compound QPX7728 (**16**, Figure 9), appears to be a promising agent for use in combination with a β-lactam antibiotics for the treatment of a wide range of multidrug-resistant Gram-negative bacteria [98]. The clinical development of QPX7728 (**16**, Figure 9) is still in progress (Phase III Clinical Trials) [99]. 

Addressing the need for effective oral therapies to treat Gram-negative bacterial infections, efforts were directed toward identifying an oral prodrug of the β-lactamase inhibitor clinical candidate QPX7728 (**16**, Figure 9). From the 17 prodrugs synthesized, compound QPX7831 (**17**, Figure 9) emerged with optimal properties across all key attributes—rates of cleavage to the active form in vitro, pharmacokinetics across species, and crystallinity [100]. 

**Figure 10:**
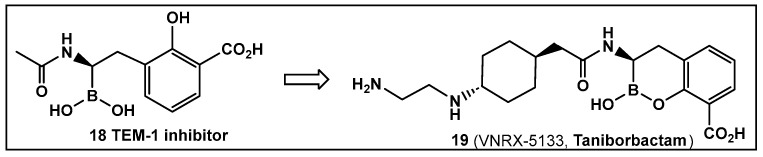
Hemiboronic acid **19** VNRX-5133, Taniborbactam), a pan-β-lactamase inhibitor developed based on the binding of the boronic acid **18** as TEM-1 inhibitor [101,102].

Another direction in the evolution of the hemiboronic acids as broad-spectrum inhibitors led to the synthesis of Taniborbactam (VNRX-5133, **19**, Figure 10). Its design strategy is based on the amide **18** (Figure 10), which is a TEM-1 β-lactamase inhibitor [101]. The lead optimization of the narrow spectrum amide **18** (Figure 10), led to preparation of compounds with a cyclic boronate scaffold. The initial library of hemiboronic acids were weakly active, with narrower spectrum than the desired inhibitors of β-lactamase enzymes [102]. Based on the observed type of binding of this initial generation of hemiboronic acids within the β-lactamase active site, a hydrophobic group was added to enhance the van der Waals interactions between the compounds and the hydrophobic residues with the β-lactamases. This change led to VNRX-5133 (**19**, Figure 10, Taniborbactam) [102]. Taniborbactam is selective for bacterial enzymes and is nontoxic to mammalian cells, making it the first pan-spectrum b-lactamase inhibitor to enter clinical development.

Its N-(2-aminoethyl)- cyclohexylamine side chain proved to be critical for broad-spectrum β-lactamase inhibition and enhanced Gram-negative outer membrane permeability and periplasmic accumulation. Structural studies revealed that the pan-β lactamase inhibition VNRX-5133 (**19**, Figure 10) mimics the tetrahedral intermediates of both the serine-based and zinc based enzymatic hydrolysis processes [102]. 

**Figure 11:**
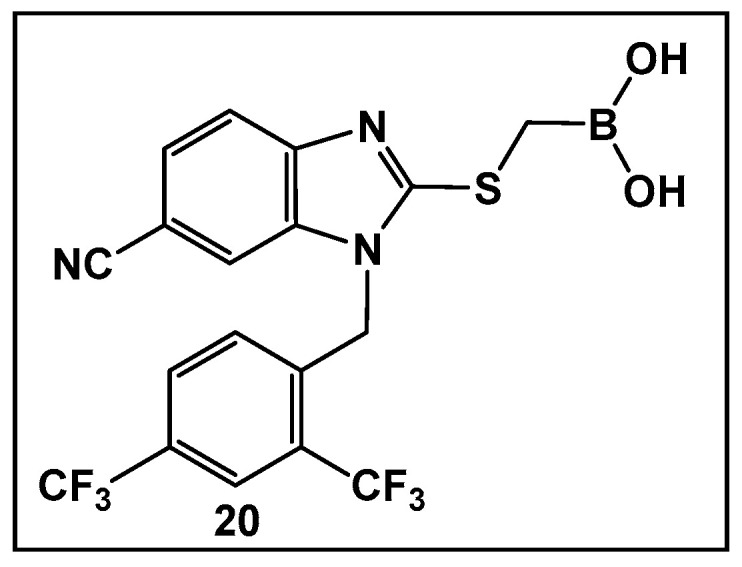
Examples of boronic acids targeting microbial defense systems: boronic acid **20** as an inhibitor of the sensor domain of BlaR in MRSA [103].

The development of boronic acids as inhibitors of other microbial defense systems against the β-lactam antibiotics, such as the sensor domain of BlaR in methicillin-resistant *S. aureus* (MRSA), has been recently reported **20** (Figure 11) [103]. Sensor domains of BlaR and/or MecR detect the presence of β-lactam antibiotics, and the information is transmitted to the cytoplasm, leading to reactivation of the antibiotic-resistance genes. Boronic acid **20** (Figure 10) is based on a benzimidazole-containing hit out of 11 million compounds screened in silico. The crystal structure confirms the engagement of **20** (Figure 10) with the serine residue of BlaR, inhibiting the BlaR induction 99% and suppressing bacterial growth by 24%. 

#### 3.1.2. Cyclic Hemiesters of Boronic Acids (Oxaboroles) as Antifungals

As the syntheses of numerous boronic acids and their esters have progressed, the versatility of hemiboronic acids as a pharmacophore has been unveiled. The FDA approval of an antifungal agent of this class validates the importance for the diversification of chemotypes in search of new alternative drug targets to aid in alleviating the current state of drug resistance [104,105,106].

**Figure 12:**
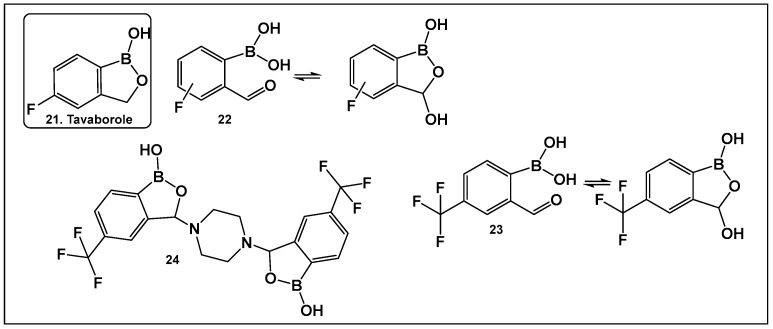
Structures of oxaboroles: FDA-approved antifungal agent Tavaborole, **21**; fluorinated derivatives of 2-formylphenyl boronic acids, **23**, **24**, designed as antibacterials and antifungals.

Tavaborole **21** (Figure 12) exerts antifungal activity by inhibiting the fungal aminoacyl-tRNA synthetase. Tavaborole binds to the diol, or terminal adenosine of the enzyme, leading to effective inhibition of fungal protein synthesis [107]. This drug (AN2690, Kerydin) was approved by the FDA in 2014 for the treatment of onychomycosis, a fungal infection of the nail [108]. The FDA approval of Vaborbactam, **9**, (Figure 7 and Figure 8), another cyclic hemiester of boronic acid, took place in 2017.

Other scaffolds, such as 2-formylphenylboronic acids **22** (Figure 12), have been evaluated for activity against the fungi *Aspergillus*, *Fusarium*, *Penicillium* and *Candida*. The *ortho* position of the formyl group is absolutely necessary for the antifungal activity of these compounds, most likely due to the presence of their tautomer (the cyclic 3-hydroxybenzooxaborole) in solution [109]. The para-fluoro analog of **22** (Figure 12), similarly to tavaborole, demonstrated the best activity against the majority of the fungi tested [109].

The effect of the replacement of fluorine with trifluoromethyl group as a substituent of the 2-formylphenylboronic **23** (Figure 12) has also been explored [110]. Both benzoxaborole and bis(benzoxaborole) derivatives of 2-formyl-4-(trifluoromethyl)phenylboronic acid have been evaluated for activity as antifungals and antimicrobials. The most potent antifungal compound is piperazine bis(benzoxaborole) **24** (Figure 12), which demonstrated MIC values of 7.8 μg/mL against *Candida albicans* and 3.9 μg/mL against *Aspergillus niger* [110]. Docking studies performed with the members in this compound library showed that binding LeuRS of *C. albicans* was analogous to that of tavaborole, **24**, (Figure 12). In addition, **23** and **24** (Figure 12) have antimicrobial activity against *Bacillus cereus* with MIC values of 7.8 μg/mL, for both compounds [110].

#### 3.1.3. AI-2 Bioisosteres as Biofilm Inhibitors: Phenyl Boronic Acids

Although originally designed as a β-lactamase inhibitor, SM23, **10** (Figure 8), has also demonstrated the inhibition of *Pseudomonas aeruginosa*, a Gram-negative microbe that is part of the ESKAPE group of pathogens [111], with biofilm production in the 0.390–25 μM range [111]. SM23, **10** (Figure 8), drastically hinders the release of the QS factors vital to *P. aeruginosa*, such as pyocyanin and elastase, and substantially downregulates the QS autoinducers. The authors report that the boronic acid derivative SM23 mimics the pathogen-produced autoinducers, downregulating both *lasI* and *lasR* gene transcripts and thus affecting the QS system, together with biofilm formation and virulence expression [111].

SM23, **10** (Figure 8), decreases the levels of biofilm formation (at 0.780–6.250 μM, about 50% of biomass reduction is observed) and that of pyoverdine production by *P. aeruginosa*, as assessed by a new in vitro model closely mimicking clinical settings (endotracheal tube contamination) [111]. It is interesting to note that even though the planktonic cells of *P. aeruginosa* strongly associate with SM23, **10**, (Figure 8), they are not affected by the compound, which might be a sign of optimism that this compound could be resistant to the known drug resistance mechanisms, in addition to its dual action as a β-lactamase and biofilm inhibitor [111].

**Figure 13:**
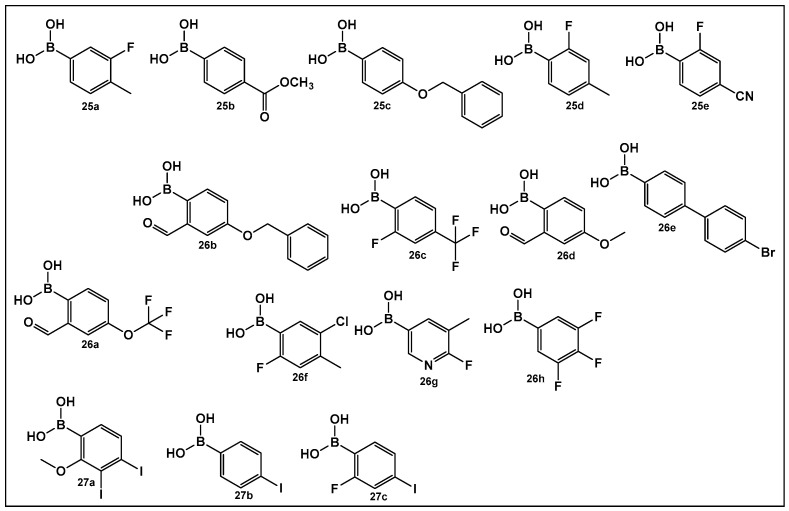
Phenyl boronic acids as QS modulators.

The activity of phenyl boronic acids, many containing mono- and poly- fluorinated or trifluoromethylated phenyl rings, belonging to different boronic acid libraries (the **25**, **26** and **27** series, Figure 13), as inhibitors of biofilm formation in *Vibrio harveyi* were reported a decade earlier than the SM23, **10** (Figure 8) [112,113]. The IC_50_ values of these inhibitors (representative examples **25**–**27** shown in Figure 13) in whole bacterial cells are in the low to submicromolar range. Five boronic acids of the **25** series (**25a**–**25e**, Figure 13) showed significant inhibitory activities, with IC_50_ in the single digit micromolar range as follows: **25a**, (IC_50_: 9 ± 5 μM); **25b**, (IC_50_: 5 ± 2 μM); **25c**, (IC_50_: 4 ± 1 μM); **25d**, (IC_50_: 4 ± 1 μM), and **25e**, (IC_50_: 6 ± 4 μM) [112]. Similarly, 11 compounds of the **26** series, representatives of which shown in Figure 13, (**26a**–**26h**) demonstrated activities at or below the single-digit range as follows: **26a**, (IC_50_: 5 ± 2 μM); **26b**, (IC_50_: 0.7 ± 0.1 μM); **26c**, (IC_50_: 2 ± 0.3 μM); **26d**, (IC_50_: 6 ± 2 μM); **26e**, (IC_50_: 3 ± 1 μM); and **26f**, (IC_50_: 6 ± 2 μM), and they also appear to not have significant inhibition of bacterial growth. At the concentrations that showed significant QS inhibition, no general cytotoxicity was observed. The rest of the compounds such as boronic acids **26g** and **26h** (Figure 13) showed IC_50_ between 11 and 100 μM, and several boronic acids compounds having IC_50_ above 100 μM [113].

A recent study has expanded the exploration of phenyl-containing halogenated acids, including three boronic acids **27a**–**27c** (Figure 13), as biofilm inhibitors of two *Vibrio* species, *V. harveyi* and *V. parahaemolyticus*, by introducing iodine into the phenyl ring [114]. The phenyl boronic acids **27b** and **27c** (Figure 13) demonstrated both antibacterial activity against *Vibrio* planktonic cells at the 100 μg/mL concentration and prevented biofilm formation in a dose-dependent manner. These two compounds prevent biofilm on the surfaces of both squid and shrimp models as well [114]. 

#### 3.1.4. AI-2 Bioisosteres as Efflux Pump Inhibitors/QS Inhibitors: Pyridine Boronic Acids

Overexpression of efflux pumps (EP) in bacteria historically has been associated with resistance to fluoroquinolone antibiotics. The established role of the EPs is the removal of substances toxic to the bacterium, such as antibiotics, metabolites, and QS compounds. In the last decade or so, a connection between the secretion of QS compounds and the EPs has been noted, based on the observation that some EPs’ inhibitors sensitize bacteria to antibiotics and can prevent biofilm formation [115,116,117,118,119,120,121,122].

**Figure 14:**
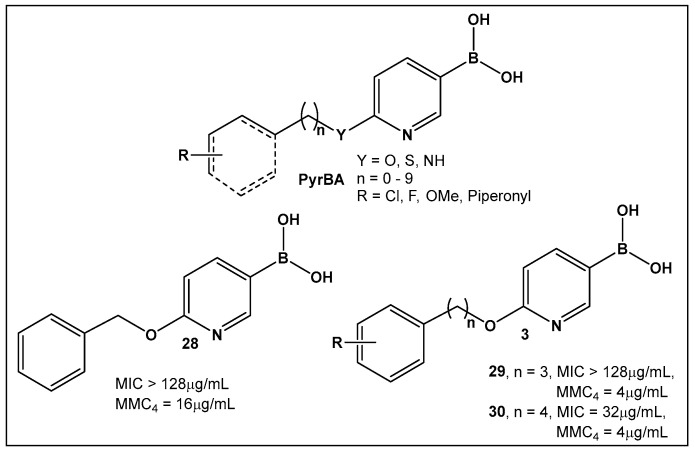
3-Pyridine boronic acid-based inhibitors of NorA efflux pump of *S. aureus*, **29** and **30**, demonstrate the best inhibitory activities and potentiate ciprofloxacin against resistant *S. aureus* by a 4-fold increase at MMC4 of 4 μg/mL.

Potential inhibitors of the NorA efflux pump of *Staphylococcus aureus* were screened using a library of 150 heterocyclic boronic acids. Those with a pyridine-3-boronic acid scaffold (PyrBA, Figure 14) were selected for further development [123,124]. The most promising compound from the initial screen shown to potentiate ciprofloxacin activity in a NorA-overexpressing SA-1199B strain (4-fold increase) was 6-benzyloxypyridine-3-boronic acid **28** (Figure 14). Further structural modification of **28** (Figure 14) led to the second-generation derivatives of pyridine-3-boronic acids, from which compounds **29** and **30** (Figure 14) showed a further 4-fold increase in activity as compared to **28** (Figure 14). These two compounds potentiate the activity of ciprofloxacin and norfloxacin, while exhibiting no significant intrinsic antibacterial activity. Importantly, they do not inhibit the mammalian P-gp efflux pump. 

**Figure 15:**
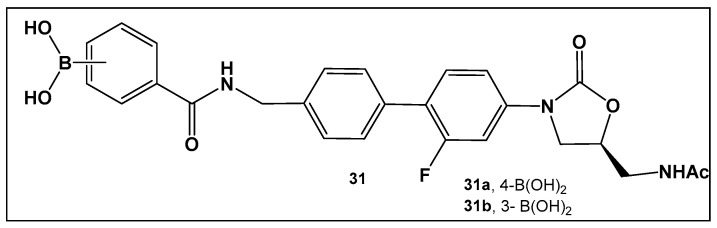
Boronic acid derivative of *N*-aryl-oxazolidinones as inhibitor in Gram-positive bacteria.

The external incorporation of phenyl boronic acid as a Lewis acid unit in the structure of oxazolidinone, **31** (Figure 15) has led to derivatives based on the *N*-aryl-oxazolidinones Linezolid and Radezolid with better activity than that of the two antimicrobials [125], with Linezolid considered as the drug of last resort against Gram-positive bacteria-induced infections [126,127,128,129]. Compounds of this type, i.e., **31** (Figure 15), have the unique binding mode of oxazolidinones structural type to the A-site pocket of the 50S subunit of bacterial ribosomes, peptidyl transferase center (PTC), negatively affecting bacterial protein synthesis and growth [130]. It is interesting to note that the Linezolid derivative, compound **31a** (Figure 15), demonstrated levels of antimicrobial activity that were eight-fold to thirty-two-fold higher than Linezolid against a panel of Gram-positive strains, and a near to one hundred-fold activity against the Gram-negative *E. coli* JW5503, a mutant strain with a defective efflux capability (MIC of 0.78 μM for **31a** vs. >50 μM for LZD), thus underscoring the potential of efflux pump incapacitation in antimicrobial/anti-biofilm drug development [125].

#### 3.1.5. AI-2 Bioisosteres as QS Modulators/Biofilm Modulators: Oxazaborolidines—Representatives of Boron–Nitrogen Bond-Containing Compounds

The first oxazaborolidines were introduced as catalysts for enantioselective synthesis since they can form chiral cations derived by various activation procedures [131,132,133,134]. These compounds can be readily obtained by the reaction of boronic acid with amino alcohols [131,132,133,134]. Several representative oxazaborolidines have been prepared and their antibacterial activity against *Streptococcus mutans* evaluated [135,136,137]. Since *S. mutans* plays a key role in dental caries and biofilm formation, identifying effective treatments against oral biofilm formation would be impactful for most of the human population.

**Figure 16:**
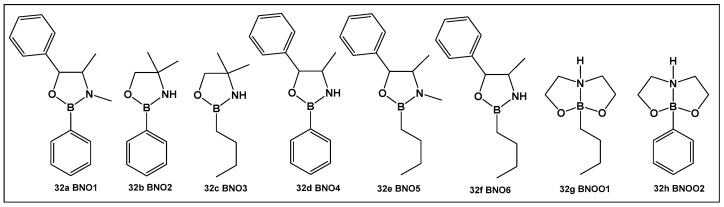
Oxazaborolidines as AI-2 bioisosteres.

From the compounds tested (**32a**–**32h**, Figure 16), the most active compound against *Streptococcus mutans* is **32e**, MIC 0.53 μM. It has alkyl groups on the nitrogen and the boron, a methyl on the nitrogen and an n-butyl on the boron, which sets it apart from the rest of the compounds. Compounds that have one of these groups but lack the others (**32a**, **32c** and **32f**, Figure 16) have lower activity (compound **32d** having the lowest MIC of 1.33 μM). In comparison with boronic acid at its maximal solubility in water (10 mM), these oxazaborolidines demonstrated antibacterial activity against *S. mutans* at much lower concentrations. When the compounds (**32a**–**32h**, Figure 16) were tested for their anti-biofilm activity, the boron–butyl moiety proved to be of great importance for the anti-adhesion effect for all the derivatives that possess it (**32c**, **32e**–**32g**, Figure 16), with diminished effect upon the incorporation of the boron atom in a fused heterocyclic ring (**32g**, Figure 16) [9,10,136]. 

Oxazaborolidines **32a**–**32e** (Figure 16), based on their structural resemblance to AI-2 QS autoinducer (Figure 4), have also been evaluated as QS modulators [138] in *V. harveyi*. Compounds **32a** and **32e** (Figure 16) strongly induced the bioluminescence in the *V. harveyi* mutant (BB170), which was lacking autoinducer 1 (AI-1), in a dose-dependent manner (0–600 μM). The latter, together with AI-2 and *V. cholerae* autoinducer 1 (CAI-1), mediates the bioluminescence of *V. harveyi*. The same two oxazaborolidines **32a** and **32e** (Figure 16) had no effect on *V. harveyi*’s (BB886) bioluminescence while lacking AI-2 [138]. Additional experiments using spent medium containing AI-2 or adding a synthetic precursor of AI-2 (termed DPD) in the presence of the compounds tested on a *V. harveyi* (MM77) mutant, which does not produce either AI-1 or AI-2, demonstrated that AI-2 is essential for the effect of **32a** and **32e** (Figure 16) on *V. harveyi*’s induction of bioluminescence. Thus, **32a** and **32e** (Figure 16) appear to be co-agonists of AI-2, since they enhance signal transduction only in the presence of AI-2 (or pre-AI-2) and only through the AI-2 cascade. That makes these two compounds the first oxazaborolidines to influence AI-2 activity [9,10,138]. 

#### 3.1.6. Aromatic Boron-Containing Heterocycles: Hemiboronic Naphthoids (i.e., Benzodiazaborines)

**Figure 17:**
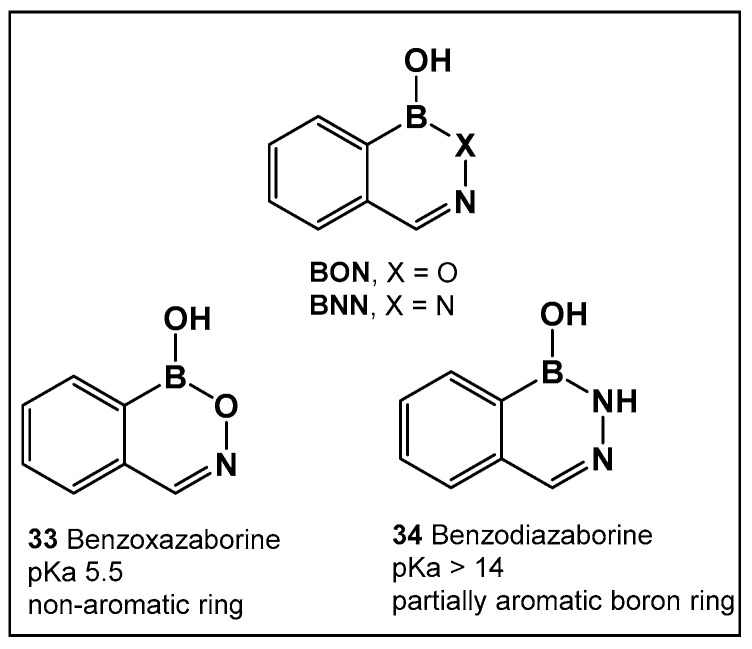
Structures of benzoxazaborines (BONs) and benzodiazaborines (BNNs) and their different chemical characteristics.

Boron compounds with a Boron–Oxygen–Nitrogen complex (B–O–N motif/BON heterocycles, Figure 17) are much less explored, even though their first representatives were prepared in the early 1960s. In recent years, there has been a growing interest in BON heterocycles as new chemotypes for drug design. The exocyclic B–O–N motif, which is readily formed under mild conditions, is unexpectedly hydrolytic and thermally stable [24,139,140].

Further exploration of the electronic proximity of boron to carbon resulted in the synthesis of a new chemical scaffold BNN (Figure 17) and the determination of its physicochemical properties [24,139,140]. While benzoxazaborines, BONs (Figure 17), with a boron-containing six-membered ring lack an appreciable aromatic character, they are chemically related to benzoxaboroles due to their moderate acidity (p*K*a 5.5 for 1a vs. 7.4 for benzoxaborole) [24]. Replacement of the oxygen with a nitrogen atom, however, leads to BNN (Figure 17), the aza-analogs, i.e., benzodiazaborines, which display partially aromatic characteristics, and a much higher pK_a_. Thus, benzodiazaborines are considered a naphthoid isostere [24,140]. Both compound classes (benzoxazaborines and benzodiazaborines) are soluble and stable in aqueous solutions, a physicochemical property that aids the biological screens [24,140]. Their differences, however, allow for these two compound classes (BON and BNN, Figure 17) to serve as functional mimics of two different “established” chemotypes, namely benzoxaboroles and aromatic scaffolds, e.g., hydroxyquinolines, naphthols, and phthalazinones, respectively. 

The 1960s marked the beginning of the exploration into benzodiazaborines as a chemotype in medicinal chemistry [141]. The antimicrobial activity of 1,2-dihydro-1 -hydroxy- 2-(organosulfonyl)benzo-, furo-, and -thieno[d][l,2,3]diazaborines have been established a decade later [142,143]. Synthesis of differently substituted benzo- and heterocyclo-substituted diazaborines and their evaluation as antimicrobials continues to draw interest [144,145,146,147]. 

**Figure 18:**
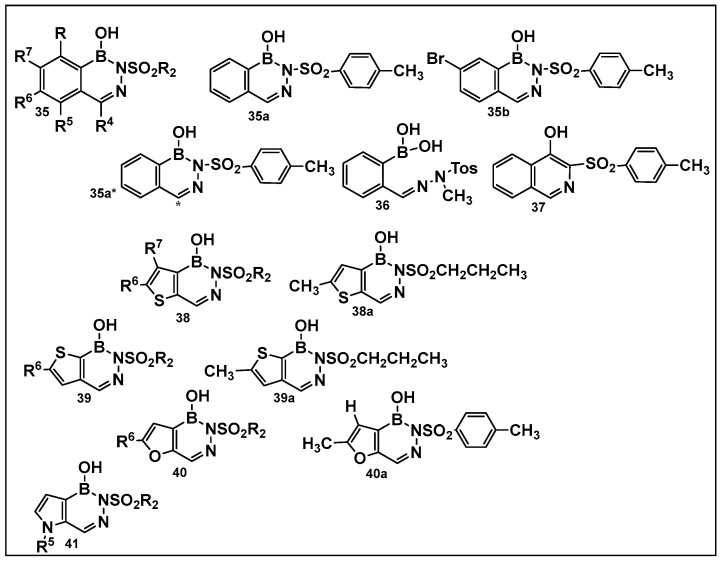
Differently substituted aromatic ring-containing diazaborines: **35**-sulfonylbenzodiazaborines,with **35a** and **35b** as the most active compound of this series against *Proteus* (12.5 μg/mL), *Klebsiella* (3.12 μg/mL)and *Salmonella* ( 6.25 μg/mL), *Neisseria gonorrhoeae* (2–8 μg/mL) and, to a lesser extent, against *Escherichia coli* (25 μg/mL and *Enterobacter* (>50 μg/mL) [144]; **38**, thieno[2,3-*d*]diazaborines are slightly more active than **39**, thieno[3, 2-*d*]-diazaborines in general; the 2-alkyl-sulfonyl derivatives of both **38** and **39** have good activities in vitro and in vivo; compound **38a** was chosen for further evaluation. *Proteus* (0.78 μg/mL), *Klebsiella* (0.39 μg/mL), and *Salmonella* (0.78 μg/mL), *N. gonorrhoeae* (1 μg/mL), *E. coli* (1.56 μg/mL), and *Enterobacter* (3.12 μg/mL); **40**, the furodiazaborines series follow a similar SAR as the 40 series; the methyl-substituted **40a** demonstrates good activity: *Proteus* (3.12 μg/mL), *Klebsiella* (1.56 μg/mL), and *Salmonella* (3.12 μg/mL), *N. gonorrhoeae* (1–8 μg/mL), *E. coli* (12.5 μg/mL), and *Enterobacter* (25 μg/mL); the pyrrolodiazaborines **41** are inactive [144]. * means radioactive-labeled compound.

**Figure 19:**
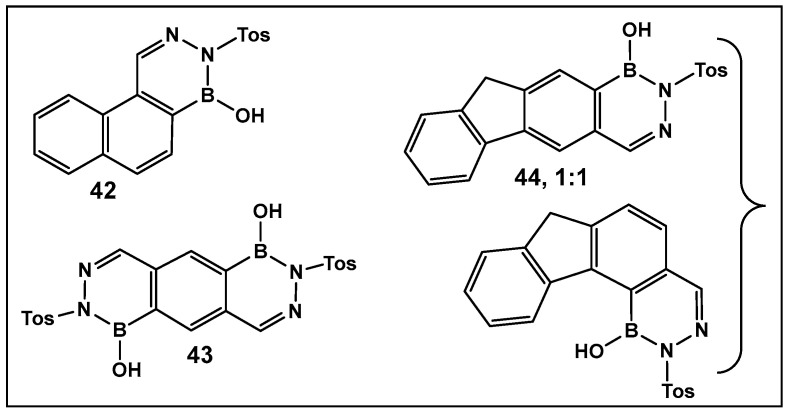
Oligocyclic diazaborine derivatives (**42**–**44**) demonstrate very low antimicrobial activity in vitro [144].

Diazaborines continue to be explored as antimicrobial agents due to their exclusive activity against Gram-negative bacteria, which are notoriously harder to treat. The diazaborine “specialization” is attributed to their well-documented mechanism of action—the inhibition of lipopolysaccharide biosynthesis in Gram-negative bacteria [141]. The SAR of derivatives of the aryl-substituted diazaborines (Figure 18) have been determined. Based on its characteristic, compound **35a** (Figure 18) has been chosen for further examination [144,145]. To address the question of whether the bicyclic diazaborines are the active antimicrobial species, the ring-opened analog **36** (Figure 18) and the isoquinoline **37** (Figure 18) were prepared as stable analogs of **35a** (Figure 15). Neither of the two derivatives (**36** and **37**, Figure 18) demonstrated antimicrobial activity similar to **35a** (Figure 18). Furthermore, neither the changes in the size or position of the aryl moiety nor the addition of a second diazaborine (compounds **42**–**44**, Figure 19) demonstrated the antimicrobial activity of their mono-aryl counterparts [144].

The radiolabeled analog of **35a**, compound 35a* (Figure 18), and compound **38a** have been further examined to determine their molecular mechanism for inhibition of *E. coli* protein EnvM [145]. Protein EnvM inhibits the enoyl-ACP and enoyl-CoA reductase activity of EnvM by binding to the protein in the presence of NAD^+^ or NADH. Based on this data, it was concluded that EnvM is the NADH-dependent enoyl-ACP reductase (EC 1.3.1.9) of *E. coli*, and proposed to rename the corresponding gene *fabI* [145]. It was also determined that the target and mechanism by which the diazaborines, using **35a** (Figure 18), inhibit the ribosome biogenesis by blocking in eukaryotic cells the large subunit formation [146].

**Figure 20:**
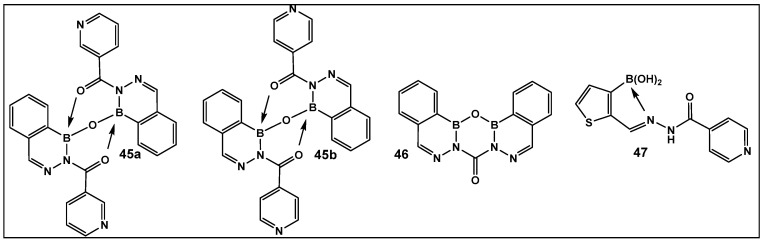
2-Acylated 2,3,1-benzodiazaborines and 2-acyl-2,3,1-diazaborine heterocycles with hydration/dehydration abilities. Compounds **45b** and **47** demonstrated the best antimicrobial activity against *Mycobacterium smegmatis* and *Escherichia coli*, with MIC values of 2–32 (μg/mL) [147].

The 2-Acylated benzodiazaborines as well as the heterocyclo-diazaborines (Figure 20) were also prepared and their antimicrobial activity evaluated [147]. From the prepared library of compounds, several demonstrated activity against *E. coli*. Two others were active against *Mycobacterium smegmatis* (**45b** and **47**, Figure 20). The finding that these two compounds have isoniazid covalently embedded in their structures suggests that they might be acting as isoniazid prodrugs for *M. smegmatis* [147].

**Figure 21:**
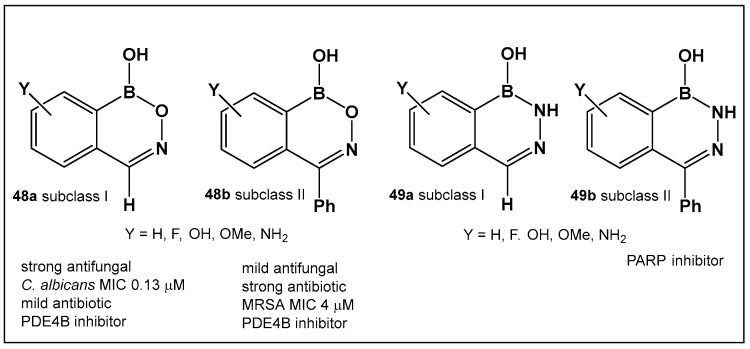
Structures of benzoxazaborines **48** (BONs) and benzodiazaborines **49** (BNNs) and their different bioactivities [24,140].

The evaluation of the biological activity of benzodiazaborines (BNN, Figure 17, Figure 18, Figure 19 and Figure 20), in addition to the antimicrobial described above, has been summarized in several reports since the 1980s as inhibitors of lipopolysaccharide biosynthesis and human neutrophil elastase, as well as estrogen mimics [148,149,150]; however, that of the benzoxazaborines has remained unexplored until very recently [24,140]. A library composed of about 70 derivatives of differently substituted derivatives from both chemotypes have been characterized and screened for biological activity [106]. Differently substituted benzoxazaborines **48a** and **48b** (Figure 21) demonstrated antimicrobial activity against MRSA (MIC 4 μM) and a mild activity against *C. albicans* when the substituent on the boron-containing ring is phenyl (**48b**, Figure 21), [24]. The activity “switches” to a strong antifungal activity (*C. albicans*, MIC 0.13 μM) and mild antibiotic against MRSA (**48a**, Figure 21) in the absence of the phenyl substituent. Both subclasses (benzoxazaborines **48a** and **48b** Figure 21) are also phosphodiesterase (PDE4B) inhibitors [24]. While their benzodiazaborine counter analogs **49a** and **49b** (Figure 21) have no antimicrobial activity, they do possess anticancer activity (poly ADP ribose polymerase (PARP) inhibitors) [24].

#### 3.1.7. Dynamic Covalent Chemistry: Polymeric Structures, Hydrogels, and Photosensitizers Based on Boronic Acids

Exploration of the dynamic interconversion between boronic acids and their esters through diols (1,2- and 1,3- diols forming 5- and 6-membered esters, respectively) has been translated into the design of responsive biocompatible materials [151,152,153,154,155,156,157].

##### Boronic Acid-Containing Hydrogels—Schiff Bases Stabilized by Boronic Acids

**Figure 22:**
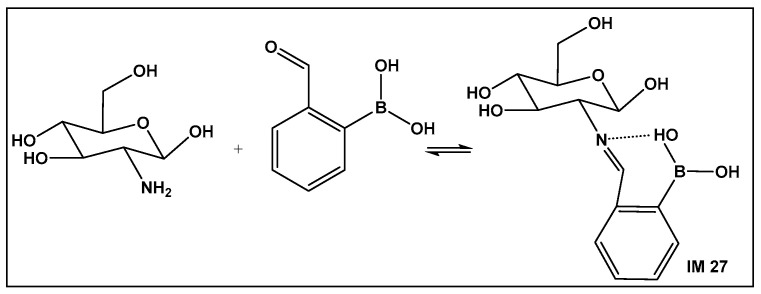
Imine–boronic acid crosslink of chitosan: a fragment depicting the amino sugar unit of chitosan reacting with 2-formyl-boronic acid to form an imine IM **27**.

Recent approaches for wound healing include the design of moldable, self-healing hydrogels. The latter are three-dimensional polymeric networks, considered highly biocompatible due to their high degree of hydration [158,159,160,161,162,163,164,165,166,167,168]. One of the most common drawbacks of the traditional wound dressing, as well as the traditional hydrogel dressing, is that they are made of relatively rigid materials that keep them somewhat embedded in the wound throughout the healing process. Removal of these dressings can trigger further pain, as well as tissue damage hence the need for self-removable/degradable materials. One of the natural polymers, chitosan, due to its physicochemical and therapeutic properties, is one of the recent examples of hydrogels that utilize the reversibility of the boronic esters under physiological conditions. In addition, chitosan has intrinsic antimicrobial and fungicidal activity [163]. Crosslinking of the natural polymer to prepare a hydrogel can be accomplished via chemical or physical means. The former gives greater stability of the crosslinked structure. It involves imine formation from the primary amino groups of the chitosan with dialdehydes (e.g., glutaraldehyde). The imine bond is easily hydrolysable, which makes the hydrogels responsive to changes in pH and biodegradable. The toxicity of dialdehydes, however, restricts their use for biomedical applications. Replacement of the dialdehydes with 2-formyl boronic acids has led to new chitosan–boron-containing crosslinks, where the boronic acid helps stabilize the imine (IM **27**; Figure 22) formed from the aldehyde moiety of the boronic acid and the amine moiety of the chitosan, i.e., imino–boronate formation (Figure 22). The imino–boronate formation has been also employed for construction of hydrogels as drug delivery systems [164], and it is an established strategy in preparation of bioconjugates and kinase inhibitors [165,166]. The chitosan crosslinked via imine, stabilized by the boronic acid (Figure 22) hydrogel, demonstrated fungicidal activity at a low concentration of 0.142% of 2-formyl boronic acid in hydrogel against *C. albicans* and *C. glabrata* both in their planktonic state. In addition, the inhibition of the metabolic activity of the corresponding *Candida* biofilms by more than 99.5% occurred, which makes these hydrogels promising candidates for the treatment of vulvovaginitis infections [162].

Mesoporous chitosan nanofibers loaded with norfloxacin and coated with the phenylboronic acid used to aid in burn wound healing have also been reported [167].

##### Boronic Acids-Containing Nanoparticles—Exploration of Boron–Alcohol–Sugar Complexes

Bacteria can overexpress polysaccharides on their cell surface, therefore boronic acids are used to target and selectively exert an antimicrobial effect [169]. Materials capable of binding to the main components of the biofilm matrix, such as polysaccharides, can disrupt its architecture. After dispersion of the biofilm, bacterial infection can be treated by “conventional means”, e.g., antibiotics. Boronic acids’ incorporation/coating of different types of molecules from peptides to nanoparticles, designed as drug carriers, is predominately used as the affinity ligand for binding to different classes of microorganisms [170,171,172]. A library of dual-targeting micelles loaded with nitric oxide (NO) and modified with phenylboronic acid and quaternary ammonium salts against cyclodextrins has been prepared and evaluated for their antimicrobial activity against *E. coli* [171]. The idea behind the combination of the aforementioned functionalities is that the boronic acid group has the ability to interact strongly and specifically with the surface of *E. coli*, while the quaternary amine salt can interact electrostatically with bacteria, due to the bacteria’s negatively charged cell membrane. These two functionalities were hypothesized to be able to alter the molecular structure of the cell membrane as well as increase its permeability, leading to cell lysis [171]. The results showed enhanced binding and killing efficiency of this type of antimicrobial agents against *E. coli* infection [171].

Similarly, the improved binding to both the Gram-negative and Gram-positive bacteria provided by boronic acid has been utilized in the preparation of chloramphenicol-imprinted polymer particles [172]. The latter has a high antibiotic loading and a slow release of the antibiotic payload. An additional feature of such boronic acid-coated polymers to their enhanced antibacterial efficiency is their potential to act as scavengers for removal of the unused antibiotic from the environment [172].

##### Boronic Acids-Containing Photosensitizers—Exploration of Boron–Alcohol–Sugar Complexes

Exploration of the application of photodynamic therapy (PDT, where photosensitizing molecules, PS, are activated by light irradiation) has led to several strategies to obtain photo-activated microbiocidal compounds. These toxic species include reactive oxygen species (ROS), an area that has been vastly explored [173,174,175,176,177], as well as antimicrobial peptides (AMP), produced in the plant and animal kingdoms as part of their defensive strategies against pathogenic bacteria [178,179]. These approaches are relatively safe and effective and appear to not easily induce bacterial resistance, while exhibiting success in combating multidrug-resistant (MDR) bacteria [180,181,182]. However, even very effective PSs require high PS concentrations and light doses to treat biofilms [183].

Each of the approaches has drawbacks, which include the following: the light-generated ROS as a highly reactive species can damage mammalian cell components [184], and their bactericidal activity is limited by short diffusion distance of the ROS [185,186,187]; using predominately hydrophobic photosensitizers covalently bound to the AMP could have a negative effect on the AMP’s targeting efficiency [188,189]. To improve the PS’s bacteria-binding selectivity, the introduction of binding ligands with bacterial binding affinity [190,191,192,193,194] is often used to enhance the affinity of the material interfaces toward bacteria. Incorporation of boronic acids in PS, such as the use of AGA405, a silicon (IV) phthalocyanine (SiPc), has also been achieved [195]. The boronic acid–PS complex was designed to target both planktonic and biofilm-producing Gram-negative bacteria. Testing of these new PS has been performed on *E. coli* [195]. Photoactivation of 10 mm AGA405 has demonstrated significant changes in the bacterial cell membrane. Detection of the bacterial biofilm, as well as non-aggregated single bacterial cells at that concentration, has clearly demonstrated the superiority of AGA405 as compared with its counterpart, phthalocyanine, which is lacking the boronic acid substituents (Pc1). Another study using a Zn-based boronic acid-functionalized phthalocyanine, PcN4-BA, has been reported [196]. This PS demonstrated highly efficient ROS generation, leading to excellent photodynamic antimicrobial activity against the antibiotic-resistant bacterial strains [196]. The dependence of the uptake of PS and the photo-bactericidal activity in planktonic cultures and biofilms from bacterial glycans suggest an important role of the nature of polysaccharides in antimicrobial photodynamic therapy (aPDT) [195]. Still, the very elaborate organic synthesis of the aforementioned PS, covalently bound to the molecules for use as chemical weapons against bacteria, is a limiting factor for their application. While noncovalently bound biological binding agents secure more dynamic/unstable binding exist, they also suffer from a high cost. Hybrid organic–inorganic materials, termed metal−organic frameworks (MOFs), are promising photosensitizing materials [197].

Recently, a combination of boronic acid as the ligand and photosensitized porphyrin Cu(II) with one single Zr-MOF was prepared in order to enhance the MOF’s antibacterial capability by improving its binding to the saccharides on the multidrug-resistant bacterial surfaces by the boronic acids [198]. This MOF demonstrated good antibacterial activity against planktonic cells of *S. aureus*, including MRSA and *E. coli* [198]. The authors also predicted that a combination of MOF and a hydrogel as a wound dressing should have an improved controlled released and thus enhance the safety of MOF use [198].

##### Boron Clusters—Exploration of Boron-Hydrophobic Interactions Biological Membranes

Boron is less electronegative than hydrogen or carbon, therefore B–H bonds in boron cluster compounds exhibit very little polarization. Consequently, borohydrides form dihydrogen bonds [199,200], which contrasts with C–H, N–H, O–H, or S–H bonds, where the degree of bond polarization follows the electronegativity (and size) of the atom bonded to the hydrogen. As a result, the surface of the electroneutral boron clusters, and their derivatives, are hydrophobic [201,202]. Therefore, their interaction with components of biological systems is via lipid membranes and proteins [203].

**Figure 23:**
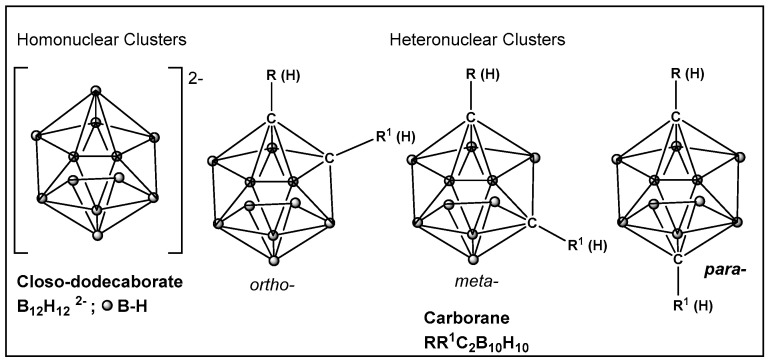
Boronic clusters—closo-dodecanoboarate and carborane.

**Figure 24:**
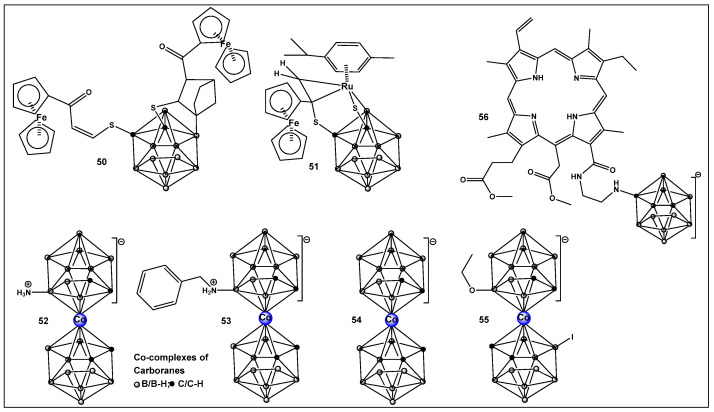
Structures of boron clusters with antimicrobial/anti-biofilm activity.

Boron clusters are another example of compounds obtained synthetically, where their ability to form bonds between their own atoms (self-catenation), similarly to carbon, has resulted in a large family of boron hydrides and heteroatom derivatives of hydrides (Figure 23). The structure of boron cluster compounds (borohydride clusters, carboranes, and metallacarboranes) have a polyhedral cage-like structure, which sets them apart from typical organic compounds’ structures that consist of chains and rings. The boron structures termed icosahedral boron cluster compounds are the most stable amongst the variety of differently shaped boron clusters. The three-dimensional aromaticity of the icosahedral boron clusters (similarly to the two-dimensional, planar aromaticity of the carbon-based aromatic rings) is responsible for their stability. The stability of the icosahedral boron clusters (i.e., **50**–**56**, Figure 24), in addition to their good bioavailability, makes them the most commonly used structures in drug design/development [203,204,205,206,207,208,209,210]. The boron clusters exploration as standalone structures or for use in conjunction with known classes of antimicrobials, e.g., β-lactams, has been on the rise since the turn of the century, and several compounds have already been identified to have antimicrobial activity against both Gram-positive and Gram-negative organisms [203]. One example is Fc2SBCp1, **50** (Figure 24), an o-carborane derivative which demonstrated activity against two multidrug-resistant (MDR) clinical isolates of *S. aureus* and *P. aeruginosa*, (with MIC and minimal bactericidal concentration (MBC) values of 36 and 72 μg/mL, respectively) [208]. Bacterial cell wall damage by Fc2SBCp1, **50** (Figure 24), resulting in cellular content leakage was observed for both bacterial species. In addition, Fc2SBCp1, **50**, (Figure 24) inhibited the biofilm formation by *S. aureus* and *P. aeruginosa* at a concentration of 0.25 × MIC (9 μg/mL), and 0.5 × MIC (18 μg/mL) of Fc_2_SBCp_1_, respectively [211].

Another ferrocene-substituted carborane, the ruthenium(II)–arene complex FcRuSB, **51** (Figure 24), evaluated by the same research group, also significantly inhibited *S. aureus* and *P. aeruginosa* biofilm formation at a concentration of 8 μg/mL [212]. Biofilm inhibition, in turn, restored the sensitivity of the two pathogens to common antibiotics, including cefuroxime sodium, penicillin G, norfloxacin, streptomycin, and roxithromycin. The mechanism of biofilm inhibition by compound **51** (Figure 24) FcRuSB was through the inhibition of extracellular matrix proteins (EMP) expression, leading to the reduced adhesion of bacteria cells. Cobalt containing boron clusters, compounds **52**–**55**, (Figure 24) were also prepared and evaluated for their anti-biofilm activity.

Compound **52**, NH_3_–[COSAN] (Figure 24) demonstrated anti-biofilm activity against Gram-positive *Staphylococcus epidermidis* and *S. aureus* and fungi (*Trichosporon cutaneum*). Its benzyl derivative, PhNH_2_-[COSAN], **53** (Figure 24) anti-biofilm activity is highly selective against *T. cutaneum* (80% inhibition of biofilm at 1 mg/mL [213]. Compound Na[COSAN] (**54**, Figure 24) demonstrated better activity than erythromycin in the inhibition of the biofilm formation of the Gram-positive *S. epidermidis*, with the best activity of 50% biofilm inhibition at a concentration of 1 mg/mL [213]. At 10 mg/mL, compound **54** (Figure 24) also has anti-biofilm formation activity against Gram-negative *E. coli* and the fungi *Candida parapsilosis* and, to a lesser extent, *T. cutaneum* (50 μg/mL) but has no effect on biofilm formation by *P. aeruginosa* [213].

The alkoxy derivative, **55**, (Figure 24) demonstrated the inhibition of MRSA biofilm formation by more than 80%, at a concentration of 0.25 × MIC (2 mg/mL), and by more than 97% at a concentration of 0.5 × MIC (4 mg/mL) thus placing compound **55** (Figure 18) among the compounds with excellent anti-biofilm activity [214].

Carboranes, have also been used in building the structure of carboranyl-chlorin e_6_, **56** (Figure 24), a potent photosensitizer for use in PDT [215], (chlorin e_6_ is an FDA-approved second-generation photosensitizer used in anticancer therapy). Compound **56** complex demonstrates considerably better photodynamic inactivation-induced bacterial cell death, as compared to the parent compound chlorin e_6_ against Gram-positive bacteria *Bacillus subtilis*, *S. aureus*, and *Mycobacterium* sp. However, neither chlorin e_6_ nor its boron derivative **56**, (Figure 24) have activity against the Gram-negative *E. coli* when illuminated at 4 J/cm^−2^. Upon increasing the illumination to 20 J/cm^−2^, only compound **56** (Figure 24) demonstrated antibacterial activity against *E. coli*, as well as against *B. subtilis*. Interestingly compound **56** (Figure 24) possesses higher antibacterial activity than chlorin e_6_ when the samples are not illuminated (i.e., dark toxicity) [215].

## 4. Summary/Outlook/Future Direction

Over the past two decades, advances in drug discovery of boron compounds have led to the development of the successful benzoxaborole drug class, the FDA-approved serine β-lactamase inhibitor (vaborbactam), and the antifungal (tavaborole). Several compounds, such as the antitrypanosomal acoziborole, antituberculosis GSK3036665, anti-MAC lung disease (*Mycobacterium avium* complex lung disease) epetraborole and the polymerase inhibitor GSK 2878175, are currently undergoing clinical trials. Examples of boron-containing antimicrobials are summarized in Table 1.

The FDA approval of boron-containing drugs represent a milestone that shows the opportunities presented by incorporation of an element that provides air-stable organic compounds with a mild Lewis acid character. The ability of benzoxaboroles, under physiological conditions, to form a variety of covalent interactions and hydrogen bonds with biological targets, similar to the boron–oxygen complexes exploited by nature, the ability to inhibit biofilm formation, and their general safety (low toxicity profile) is the basis for their success as drugs. Additionally, we expect to see an increase in research that explores the use of other boron-containing scaffolds such as the ones discussed herein. They are broadening the chemotypes of the reversible covalent binders and appear to have the ability to strike a balance between potency and selectivity in different biological systems. Covalent (irreversible) modulators of therapeutic targets have been an integral part of antimicrobial drug discovery since the discovery of Penicillin. Reversible covalent compounds provide minimization of the off-target effects. Boron-containing electrophiles, as reversible covalent modulators, represent a direction for a more tailored reactivity, which is especially important for drug development of antimicrobials. It is imperative to use drugs to which drug resistance is less likely to develop, such as those that interfere with biofilm formation.

## Figures and Tables

**Table 1:** Examples of the exploration and evolution of organoborons as antimicrobials/anti-biofilm agents from 1980s to 2024.

Entry &Ref.	Antimicrobial Activity	Chemotype/Structure	Under Clinical Development/FDA Approval
**9**. [93,94]	β-lactamase inhibitor—serine enzymes	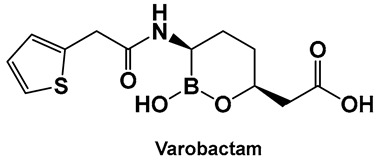 Hemiboronic acid	FDA approved (carbapenemases only, in combination with meropenem) 2017
**10**. [88,89,90,111]	β-lactamase inhibitor (incl. *Acinetobacter baumannii* cephalosporinase); anti-biofilm	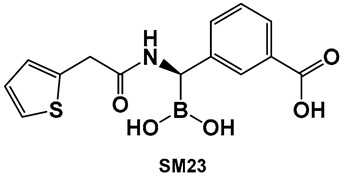 Boronic acid	
**11**. [95,96]	β-lactamase inhibitor—serine enzymes	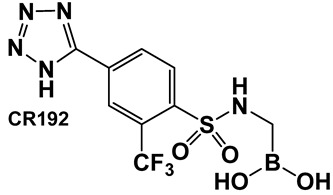 Boronic acid	
**12**. [95,96]	β-lactamase inhibitor—serine enzymes	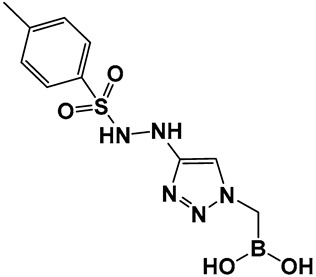 Boronic acid	
**16**. [97,98,99]	β-lactamase inhibitor—ultra-broad-spectrum serine and metallo-enzymes	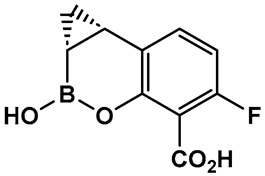 Hemiboronic acid	Under Clinical development (Phase I Clinical Trials)
**17**. [100]	β-lactamase inhibitor—ultra-broad-spectrum serine and metallo-enzymes	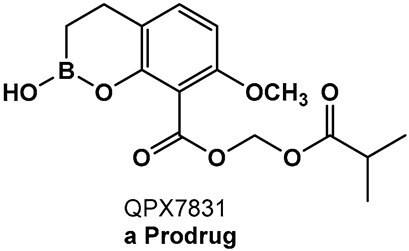 Hemiboronic acid	
**19**. [102]	β-lactamase inhibitor—pan spectrum	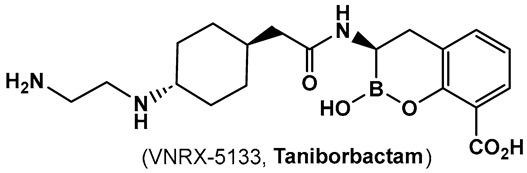 Hemiboronic acid	Under Clinical development (Phase III Clinical Trials)
**20**. [103]	Inhibitor of the sensor domain of BlaR in MRSA	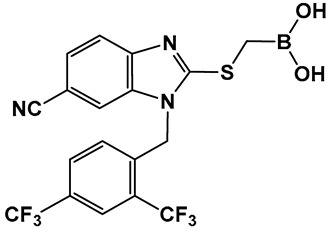 Boronic acid	
**21**. [107,108]	Antifungal activity by inhibiting the fungal aminoacyl-tRNA synthetase	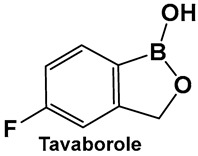 Hemiboronic acid	FDA approved for treatment of onychomycosis 2014
**25**–**27**. [112,113,114]	Inhibitors of biofilm formation in *Vibrio harveyi* (nonhalogenated/halogenated)	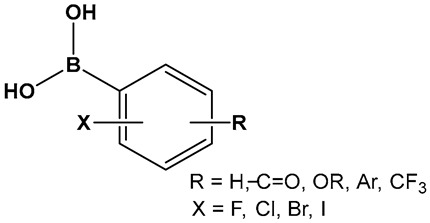 Boronic acid	
**28**. [123,124]	Bacterial efflux pump inhibitor	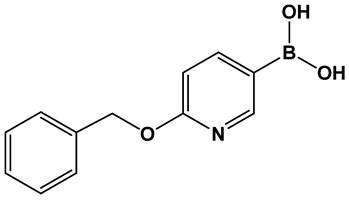 Boronic acid	
**31**. [135,136,137]	Bacterial protein synthesis inhibitor A-site pocket of the 50S subunit of bacterial ribosomes, peptidyl transferase center (PTC)	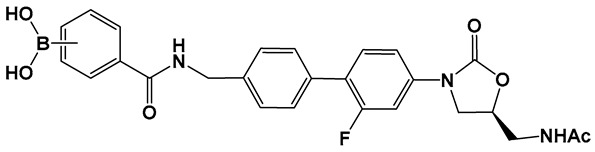 Boronic acid	
**32e**. [135,136,137]	Antimicrobial, against *Streptococcus*, anti-biofilm agent	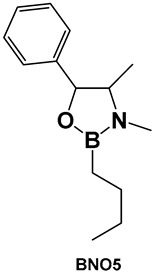 Oxazaborolidines	
**35**–**40**. [144,145,146]	Antimicrobial against *E. coli*, *Enterobacter*, *Proteus*, *Neisseria*, *Klebsiella*, *Salmonella*	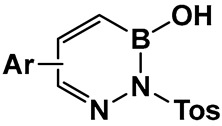 Benzodiazaborines	
**45a**, **47**. [147]	Antimicrobial activity against *E. coli* and *Mycobacterium smegmatis*	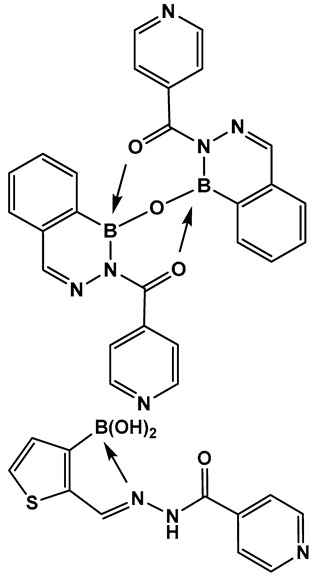 2-Acylated benzodiazaborines	
**48a**, **48b**. [24,140]	**48a**—Antifungal—*Candida* **48b**—Antimicrobial—methicillin-resistant *Staphylococcus aureus* (MRSA)	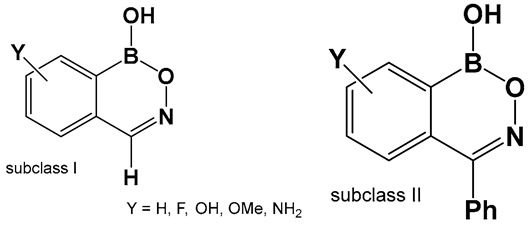 Benzoxazaborines	
**52**–**55**. [213,214]	**52**. Antimicrobial/antifungal Gram-positive *Staphylococcus epidermidis* and *S. aureus* and fungus *Trichosporon cutaneum)* **55**. Anti-biofilm MRSA	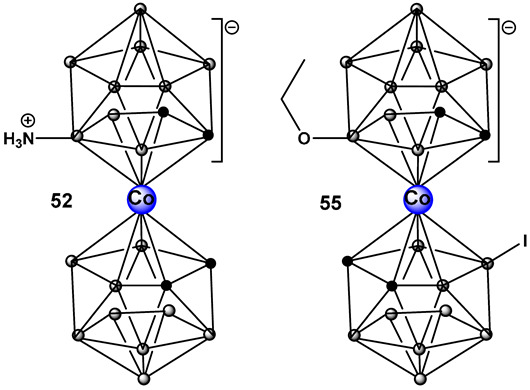 Boron clusters	

## Data Availability

Not applicable.
